# The Principles and Practice of Obstetrics

**Published:** 1863-02

**Authors:** 


THE PRINCIPLES AND PRACTICE OF OBSTETRICS. BY GUNNING S. BEDFORD, A.M., M.D., Professor of Obstetrics, &c., University of New York. *Third edition.*

In four months after the first edition of the above book, the author was called upon for the second, and now, thirteen months from the first edition, is issued the third. No more significant expression of approbation from the profession could be desired and, certainly, more reasonably expected than these facts. The author who can issue the third edition in this length of time, as the result of a demand for his book, is fortunate. The cause of this unprecedented sale of Dr. BEDFORD'S obsterics is sufficiently obvious to any person who will examine the work. It is a plain, exact, practical replete system of midwifery, brought up to the present times. While it is full, in extent and practical detail, it is not encumbered with unnecessary disquisitions and verbiage. This makes it a good book for students, an excellent work for busy practitioners, and a faithful record or mark by which the historian of obstetric medicine may be guided in his estimation of its advance and status at the present day. "Of making books there is no end," is, perhaps, more true now than it ever was before, but the art of book-making is not any better understood than in former times. Men's judgment as to the merits of books, however, is becoming better all the time, hence, the literary and scientific lumber that lies in mouldering heaps in libraries, libraries without blot or soil, while there are so few well-thumbed and use-worn volumes. Among the latter, will stand, for many years, this practical work of Dr. BEDFORD. There is now no book in print on the subject of obstetrics, that we can recommend before BEDFORD'S; and we earnestly hope that the author may long live to keep it posted up to the times, as the science advances.

